# Civil Service Examination, New York City

**Published:** 1895-11

**Authors:** 


### CIVIL SERVICE EXAMINATION, NEW YORK CITY.

THE following civil service examination questions were asked before the New York City Board for the Street Cleaning Department Veterinary Staff:

1. What is a contagious miasmatic and miasmatic contagious disease? 2. What are the physical signs of lobar pneumonia in the second stage? 3. What diseases are glanders liable to be mistaken for? 4. Where are exostoses usually found, and the causes? 5. Give the differential diagnosis between azoturia and spinal meningitis. 6. Differentiate between cold, warm, and intermittent lameness. 7. Describe periodic ophthalmia. 8. Describe the symptoms of dislocation of patella. 9. What are the indications of treatment for cartilaginous quittor? 10. Differentiate between spasmodic and flatulent colic. 11. Give characteristic symptoms of glanders. 12. Where are abscesses usually found? 13. What is the best method of stopping hemorrhage of the palatine? 14. What effect has mallein upon an animal? 15. What are the sanitary indications in case of an outbreak of glanders in a large stable of horses? 16. What are the indications of treatment in punctured wounds of the feet? 17. Differentiate between a fracture and dislocation. 18. What are the causes and symptoms of subcutaneous emphysema? 19. What are the indications of tracheotomy? 20. What are the causes of urticaria?

